# Traumatic injury mortality prediction (TRIMP-ICDX): A new comprehensive evaluation model according to the ICD-10-CM codes

**DOI:** 10.1097/MD.0000000000029714

**Published:** 2022-08-05

**Authors:** Guohu Zhang, Muding Wang, Degang Cong, Yunji Zeng, Wenhui Fan

**Affiliations:** a Department of Emergency Intensive Care Unit; b Department of Emergency Medicine; c Department of Thoracic Surgery; d Department of Orthopedic, Affiliated Hospital of Hangzhou Normal University, Hangzhou, Zhejiang, PR China.

## Abstract

Various assessment methods based on the International Classification of Diseases, Tenth Edition, Clinical Modification (ICD-10-CM), such as ICD-10-CM Injury Severity Score (ICISS), trauma mortality prediction model (TMPM-ICD10), and injury mortality prediction (IMP-ICDX), are purely anatomic trauma assessment, which need to be further improved. Traumatic injury mortality prediction (TRIMP-ICDX) is a comprehensive assessment method based on anatomic injuries and incorporating available information to determine whether it is superior to Trauma and Injury Severity Score (TRISS) and IMP-ICDX in predicting trauma outcomes. This retrospective cohort study was based on data from 704,287 trauma patients admitted to 710 trauma centers in the National Trauma Data Bank of the United States in 2016. The TRIMP-ICDX was established using anatomical injury, physiological reserves, and physiological response indicators. Its performance was compared with the IMP-ICDX and TRISS by examining the area under the receiver operating characteristic curve (AUC), calibration (Hosmer-Lemeshow goodness-of-fit test, HL), and the Akaike information criterion (AIC). The TRIMP-ICDX showed significantly better discrimination (AUC_TRIMP-ICDX_ 0.968; 95% confidence interval (CI), 0.966–0.970, AUC_TRISS_ 0.922; 95% CI, 0.918–0.925, and AUC_IMP-ICDX_ 0.894; 95% CI, 0.890–0.899), better calibration (HL_TRIMP-ICDX_ 5.6; 95% CI, 3.0–8.0, HL_TRISS_ 72.7; 95% CI, 38.4–104.5, and HL_IMP-ICDX_ 53.1; 95% CI, 26.6–77.8), and a lower AIC (AIC_TRIMP-ICDX_ 24,774, AIC_TRISS_ 30,753, and AIC_IMP-ICDX_ 32,780) compared with TRISS and IMP-ICDX. Similar results were found in statistical comparisons among different body regions. As a comprehensive evaluation method based on the ICD-10-CM lexicon TRIMP-ICDX is significantly better than IMP-ICDX and TRISS with respect to both discriminative power and calibration. The TRIMP-ICDX should become a research method for the comprehensive evaluation of trauma severity.

## 1. Introduction

Trauma scoring is based on the International Classification of Diseases, Ninth Edition, Clinical Modification (ICD-9-CM) codes, such as the ICD-9-CM injury severity score (ICISS) and the trauma mortality prediction model (TMPM-ICD9).^[1,2]^ The TMPM-ICD9 has been reported to be more efficient than the ICISS in predicting death outcomes, but both of them are solely anatomy-based injury severity scoring methods. In the modified version of the trauma and injury severity score (TRISS) system, the injury severity score (ISS) is replaced by ICISS; other variables include age, Glasgow Coma Scale (GCS) score, systolic blood pressure (SBP), and respiration rate (RR). Its discriminative ability is superior to that of the original TRISS system.^[3]^ The definition of ISS is as follows: firstly turn the original 9 body regions (BR) into 6 predefined BRs, then take the square sum of the 3 most severe Abbreviated Injury Scale (AIS, its score ranges from 1 to 6, with 1 being a minor injury and 6 being a currently incurable injury) codes in the 6 BRs as the ISS score.^[4]^ Therefore, ISS inevitably has congenital defects which may affects the results of TRISS. The Harborview Assessment for Risk of Mortality (HARM) is a comprehensive scoring method which is based on ICD-9-CM.^[5]^ It has 80 variables, including age, injury mechanism, injury categories, and the comorbidy conditions, etc Although its predictive ability is higher than TRISS and ICISS, HARM is difficult for clinicians to follow.

Most countries currently use the ICD-10-CM code as a common diagnostic code, in this case, ICD-9-CM will become history. In trauma assessment which is based on the ICD-10-CM code, the scoring methods purely based on anatomical injury include ICISS, TMPM-ICD10, and injury mortality prediction (IMP-ICDX).^[6–9]^ In order to improve discriminative ability, variables such as age, gender, and injury mechanism in the assessment are added into IMP-ICDX. However, it still inadequately reflects the contribution of other available information (such as vital signs, GCS, etc).

Considering accuracy and differences in trauma assessment methods, accurate prediction of trauma outcomes can guide clinicians to allocate medical resources effectively, which benefits the prognosis and treatment of patients. A comprehensive assessment based on the severity of anatomical injury was developed, incorporating physiological reserve (such as age, gender, and the sum of comorbid weighted indexes (SCWI)) and response to injury (including vital signs, GCS score, admission to intensive care unit (ICU), etc). In the proposed approach, we call it traumatic injury mortality prediction (TRIMP-ICDX), the models were compared with respect to their performance. The goal of this study is to confirm that predicting death or survival from trauma by using TRIMP-ICDX more accurate than by using TRISS and IMP-ICDX.

## 2. Materials and Methods

### 2.1. Data source

This retrospective cohort study was conducted on 704,287 patients hospitalized with traumatic injuries in 2016 from the National Trauma Data Bank (NTDB) data in the United States.^[10]^ Available information included patient demographics, ICD-10-CM diagnostic and injury codes (the clinical modification developed by the United States), ISS (version 2005), SBP, RR, heart rate, GCS score, mechanism of injury (based on ICD-10-CM E-codes), SCWI, ICU admission, the total number of days on mechanical ventilation, surgical operation, in-hospital mortality, and encrypted hospital identifiers. The dataset included 876,413 patients with 1 or more ICD-10-CM diagnostic and injury codes.

The following patients were excluded from our analysis: patients aged <1 year (3287), or more than 89 years (51,631); patients with nontraumatic diagnoses (e.g., drowning, suffocation, and poisoning) or burns (40,493), missing or invalid data (missing data on the length of hospital stay or gender) (26,019), missing data on vital signs (28,439); patients who were transferred to another facility (32,833) or dead on arrival at hospitals (9229); and patients who sustained a single injury or multiple injuries and with an ISS score of -2 (3660). E-codes were mapped to 1 of the 6 mechanisms of injury: fall, motor vehicle crash, blunt injury, violence, stabbing, and firearm wound. The final dataset consisted of 704,287 patients admitted to 710 hospitals.

### 2.2. Comorbidity

In this study, the sum of comorbid weighted indexes (SCWI) was used as an auxiliary index for comorbidity diseases,^[11]^ which could improve the prediction result after a patient getting injured compared with the well-known and recognized Charlson Comorbidy Index (CCI).^[12]^

### 2.3. Development overview of TRIMP-ICDX

In this study, a total of two-thirds of the data (the TRIMP-ICDX development dataset) were used to assess the regression coefficients of TRIMP-ICDX. The corresponding coded values were set based on the mortality rate of each available variable, which are shown in Supplemental Digital Content (Appendix 1, http://links.lww.com/MD/G970). We mainly adopted the severity of anatomical injury, physiological reserve, and physiological response indicators to trauma as fundamental predictors to establish a separate logistic regression model. Fractional polynomial analysis was recommended for continuous variables (e.g., NBR and age).^[13]^ Variables with or without binary indicators (such as mechanical ventilation, ICU admission, etc) were expressed as 1 or 0. The corresponding coefficients of the logistic regression variables are listed in Table 3, and the deduced specific formula for TRIMP-ICDX is presented in Supplemental Digital Content (Appendix 2, http://links.lww.com/MD/G970). The remaining one-third of the data (the internal validation dataset) was adopted to estimate the statistical performance of the TRIMP-ICDX, TRISS, and IMP-ICDX models, and to compare the results at different body regions. This classification of 7 BRs (1, Head and neck; 2, Face; 3, Chest; 4, Abdomen; 5, Extremities; 6, Skin and 7, Other) in this study and method for calculating IMP-ICDX were developed based on Wang et al^[9]^ whereas that of TRISS was based on Champion et al and Javali et al.^[14,15]^

### 2.4. Statistical analysis

The statistical tests used in this text were the area under the receiver operating characteristic curve (AUC), the Hosmer-Lemeshow (HL) goodness-of-fit test, and the Akaike information criterion (AIC). A lower AIC indicated a better model. The bias-corrected 95% confidence intervals for AUC and HL were calculated using the bootstrapping algorithm (1000 replications). *P*-value < 0.05 was considered statistically significant. All statistical analyses were performed using STATA/MP version 14.0 for Windows.

## 3. Results

**Figure 1.**
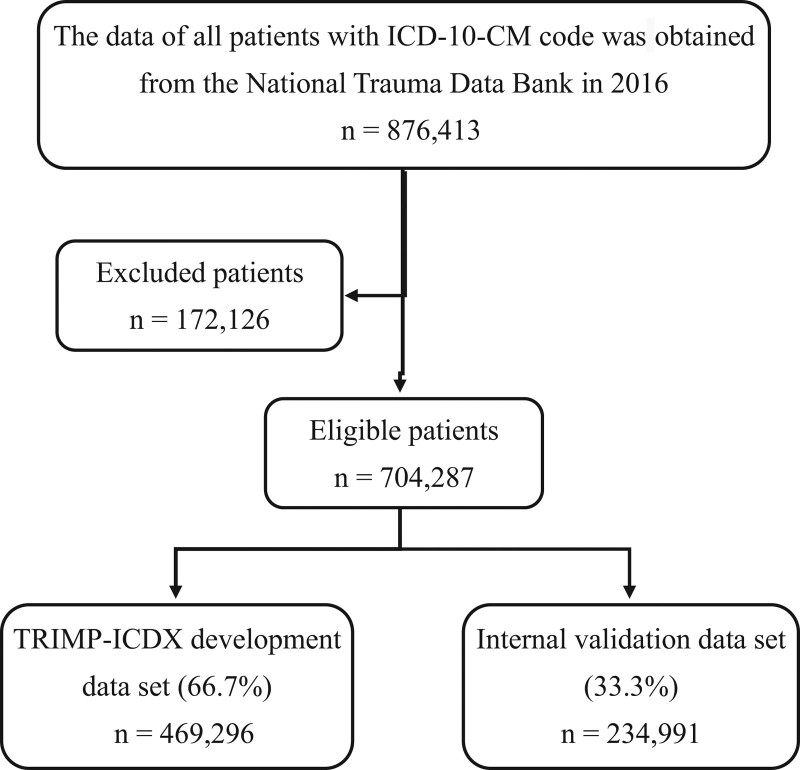
TRIMP-ICDX Traumatic injury mortality prediction for ICD-10-CM code.

The demographic data of patients with blunt injuries and penetrating injuries are summarized in Table 1. In this article, the overall mortality rate was 2.35%. The median age of our cohort was 49 (26–69). Females comprised 38.5% of the patients. Most patients were admitted to a Level I or II trauma center (89.5%). 71.3% was Whites and 14.7% was Blacks. Fall (43.9%) and motor vehicle accidents (36.5%) were identified as the most common causes of trauma. Recruitment details are shown in Figure 1.

**Table 1:** Characteristics of patients

Variables	Blunt injury (n = 619,241)	Penetrating injury (n = 85,046)
Age, median (IQR), y	52 (28–71)	30 (21–46)
Gender, female (%)	256,421 (41.4)	14,870 (17.5)
Trauma center designation (%)*		
I	329,652 (53.3)	54,065 (63.5)
II	222,401 (35,9)	24,503 (28.8)
III	54,534 (8.8)	4590 (5.4)
IV	2544 (0.4)	220 (0.3)
Not applicable	10,110 (1.6)	1668 (2.0)
ISS, mean†	8.97 (7.80)	7.80 (8.85)
LOS, days, mean	5.16 (7.70)	4.73 (8.35)
SBP (mm Hg), mean	138.1 (26.45)	133.4 (25.31)
RR (breaths/min), mean	18.72 (4.54)	18.79 (4.91)
HR (beats/min), mean	88.5 (19.95)	91.0 (21.47)
GCS, mean	14.31 (2.34)	14.10 (2.79)
NBR, mean	3.56 (3.15)	3.27 (2.54)
SCWI, mean	1.49 (1.67)	0.98 (1.23)
Died (%)	13,926 (2.25)	2657 (3.12)

The statistical performance of the 3 models is presented in Table 2. Compared with the IMP-ICDX and TRISS models, TRIMP-ICDX exhibited superior discriminative ability, calibration, and AIC statistic to a significant degree. The statistical results are similar when comparing different BRs. The regression coefficients of TRIMP-ICDX are listed in Table 3.

**Table 2:** Outcome statistics of 3 models in different body regions.

Model description	BR	N	AUC (95%CI)	HL stat	AIC
TRISS	All	216,330	0.922 (0.918–0.925)	72.73	30,752.7
	1	72,528	0.932 (0.928–0.936)	64.29	16,682.4
	2	15,008	0.889 (0.842–0.936)	9.20	609.1
	3	38,165	0.871 (0.858–0.884)	15.01	5826.1
	4	14,046	0.914 (0.899–0.928)	18.74	2182.1
	5	74,222	0.800 (0.784–0.816)	24.56	5047.0
	6	1213	0.935 (0.888–0.983)	0.97	136.0
	7	1148	0.972 (0.947–0.997)	1.54	55.0
IMP-ICDX	All	234,991	0.894 (0.890–0.899)	53.00	32,779.5
	1	77,899	0.908 (0.903–0.914)	64.25	17,133.7
	2	16,488	0.634 (0.560–0.708)	2.99	788.9
	3	38,961	0.810 (0.793–0.827)	9.45	6611.0
	4	15,345	0.897 (0.881–0.914)	14.25	2415.7
	5	83,779	0.791 (0.773–0.808)	11.56	5492.9
	6	1305	0.855 (0.762–0.947)	35.14	191.8
	7	1214	0.575 (0.315–0.835)	3.48	88.5
TRIMP-ICDX	All	234,991	0.968 (0.966–0.970)	5.58	24,773.6
	1	77,899	0.969 (0.967–0.971)	4.39	12,383.0
	2	16,488	0.966 (0.951–0.980)	1.00	497.5
	3	38,961	0.947 (0.941–0.953)	4.65	4884.5
	4	15,345	0.958 (0.951–0.966)	10.31	1891.7
	5	83,779	0.943 (0.935–0.951)	5.84	4033.7
	6	1305	0.956 (0.932–0.979)	0.79	139.4
	7	1214	0.988 (0.971–1.004)	0.14	39.7

**Table 3:** TRIMP-ICDX regression coefficients

Predictor		Coefficients	Robust std. error	*Z*	*P* > *z*	95% CI
WMDP_1_	C_1_	1.35233	0.16729	8.084	0.000	1.02443 − 1.68022
WMDP_2_	C_2_	1.39599	0.14267	9.785	0.000	1.11636–1.67563
WMDP_3_	C_3_	0.72753	0.10727	6.782	0.000	0.51729–0.93778
WMDP_4_	C_4_	0.43396	0.09546	4.546	0.000	0.24686–0.62105
WMDP_5_	C_5_	0.57471	0.08805	6.527	0.000	0.40212–0.74730
WMDP_1_^3^	C_6_	0.34710	0.03897	8.906	0.000	0.27071–0.42348
WMDP_2_^3^	C_7_	–0.98051	0.08345	11.750	0.000	–1.14407 to –0.81696
WMDP_3_^3^	C_8_	–0.24364	0.04355	5.595	0.000	–0.32899 to –0.15829
WMDP_1_ × WMDP_2_	C_9_	0.14646	0.01030	14.217	0.000	0.12627–0.16665
Same region	C_10_	–0.14674	0.03118	4.706	0.000	–0.20786 to –0.08563
NBR	C_11_	0.03461	0.00787	4.400	0.000	0.01919–0.05002
Ln(NBR)*	C_12_	–0.90484	0.09009	10.044	0.000	–1.08142 to –0.72826
Age^3^	C_13_	2.17 × 10–^5^	2.12 × 10–^6^	10.232	0.000	1.75 × 10–^5^–2.58 × 10–^5^
Ln(age) × age^3^	C_14_	–3.79 × 10–^6^	4.69 × 10–^7^	8.082	0.000	–4.71 × 10–^6^ to –2.87 × 10^-6^
Gender	C_15_	0.23173	0.02820	8.219	0.000	0.17647–0.28700
SCWI	C_16_	0.17533	0.00681	25.759	0.000	0.16199–0.18867
ICU admission	C_17_	0.45740	0.03969	11.523	0.000	0.37959–0.53520
Ventilator	C_18_	2.06323	0.03912	52.739	0.000	1.98655–2.13991
Operation	C_19_	0.46088	0.03315	13.902	0.000	0.39590–0.52586
Injury mechanism	C_20_	0.20469	0.01466	13.961	0.000	0.17959–0.23342
GCS	C_21_	–0.11458	0.00325	35.299	0.000	–0.12029 to -0.10821
SBP	C_22_	0.40317	0.01372	29.382	0.000	0.37627–0.43006
Heart rate	C_23_	0.25384	0.01305	19.455	0.000	0.22827–0.27941
RR	C_24_	0.10264	0.01142	8.985	0.000	0.08025–0.12503
Constant	C_0_	–9.16790	0.14341	63.930	0.000	–9.44897 to –8.88682

**Figure 2.**
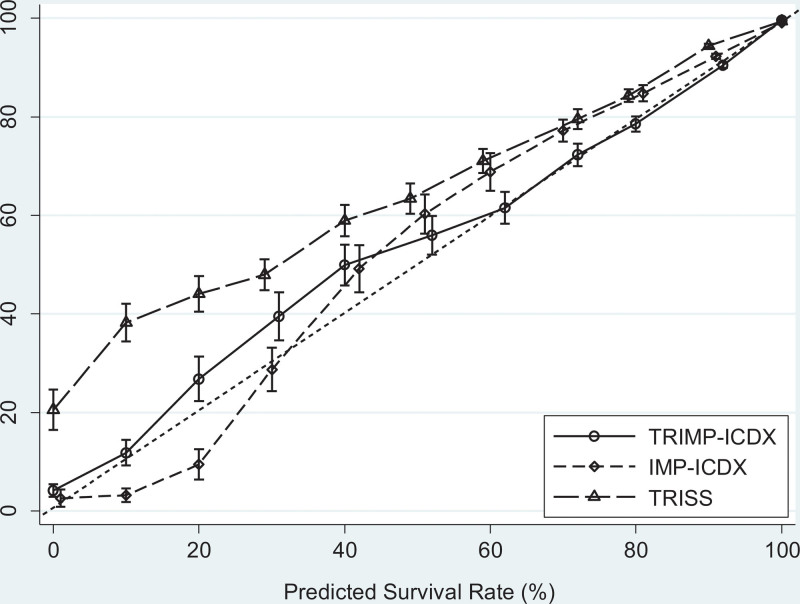
The dotted line indicates perfect calibration. All 3 models had 95% binomial confidence intervals based on the same validation dataset of 234,991 patients. TRIMP-ICDX Traumatic injury mortality prediction for ICD-10-CM, IMP-ICDX Injury mortality prediction for ICD-10-CM, TRISS Trauma and injury severity score.

Figure 2 shows the superiority of TRIMP-ICDX calibration to IMP-ICDX and TRISS calibrations. The survival rate of TRIMP-ICDX is uniformly distributed near the perfect reference line, the TRISS survival rate distribution is above the perfect reference line, and the survival curve of IMP-ICDX exhibits an inverted S-curve.

## 4. Discussion

Various assessment methods based on the ICD-9-CM code, such as the original ICISS, TMPM-ICD9, TRISS, and HARM, among others, have rarely been used.^[1–3,5]^ The ICISS, TMPM-ICD10, and IMP-ICDX, which are based on the ICD-10-CM code, are assessment techniques that are purely based on anatomical trauma.^[6–9]^ The IMP-ICDX evaluated using 3 logistic regressions mutual modifications show more accurate prediction results than those of TMPM-ICD10 with a single regression model. Meanwhile, IMP-ICDX has improved its discriminative ability with the incorporation of age, gender, and injury mechanism. But IMP-ICDX does not fully use clinically available information, such as GCS, endotracheal intubation, SCWI, and so on.

The data in this research were derived from NTDB in 2016,^[10]^ which presents a description list of ICD-10-CM codes for trauma. The NTDB is the largest and most reliable publicly available database worldwide with more representative data from different regions and trauma centers in the United States. The TRIMP-ICDX in this study was based on the severity of anatomical injury, incorporating more than 10 auxiliary variables with statistical significance (physiological reserve indicators, such as age, gender, and SCWI; physiological response indicators, such as SBP, RR, GCS, ICU admission, need for mechanical ventilation or emergency surgery, etc) to create a logistic regression model. The discriminative ability and calibrations of survival and nonsurvival were superior to those of IMP-ICDX and TRISS. The statistical results were similar for different BRs (Table 2). The specific calculation formula is given in Supplemental Digital Content (Appendix 2, http://links.lww.com/MD/G970).

The data used in this study were from patients of all ages (from 1 to 89 years old). However, TRISS is only used for patients older than 14 years,^[16,17]^ which may not be appropriate for trauma patients younger than 15 years old. When gender, SCWI, and children under 15 years old were included in the physiological reserve, the prediction results of TRIMP-ICDX were improved. The SCWI can be used as an independent factor to predict the probability of traumatic death.^[12]^ The mechanism of injury and the need for emergency surgery can be understood as indirect indicators of physiological response. Simultaneously, compared with fixed categories, nonparametric regression more accurately explained the relationship between age and traumatic death,^[12,18]^ that is, there is no grouping of age, and the results should be better. Increasing auxiliary variables, such as whether to stay in ICU or whether mechanical ventilation is needed can help to predict the outcome of trauma.^[9]^

Clinically, there are several indications for patients entering the ICU after injury, such as further life support after cardiopulmonary resuscitation, monitoring and treatment after severe trauma, patients requiring mechanical ventilation, etc Also, there are indications for mechanical ventilation after trauma. For example, patients with postinjury disturbance of consciousness or loss of spontaneous breathing after injury. In general, it is patients with severe injured that require mechanical ventilation and/or admission to the ICU, which can also be regarded as an indicator of indirect physiological response to trauma. This has been confirmed in the existing literature: such as IMP-ICDX.^[9]^ The possibility of death or survival can be assessed in trauma patients who can be diagnosed clearly after admission.

Based on the ISS, TRISS incorporates GCS, SBP, RR, and age in the evaluation of the survival probability of patients.^[16]^ Updated several times, the latest version of TRISS was published in 2011.^[17]^ Compared with ISS, TRISS predicts outcomes more accurately. This study showed that the lowest survival rate of TRISS exceeded 20%, which is significantly higher than that of TRIMP-ICDX. Probably influenced by ISS, the survival prediction curve of TRISS is above the perfect reference line (Fig. 2).

HARM is based on 80 available variables, such as anatomical indicators and physiological reserves, and is evaluated by independent logistic regression. A study found that compared with TRISS and ICISS, HARM more accurately predicted survival or death from trauma^[5]^; however, it did not take full advantage of statistically significant physiological response indicators, such as vital signs, mechanical ventilation, and whether surgery was required. This article only used more than 10 available variables, making it easier for clinicians to evaluate the results. The absolute AUC value of TRIMP-ICDX is better than HARM (0.968 vs 0.958).

This study evaluated the overall sample of patients described by the ICD-10-CM code. Cases of blunt injuries or penetrating injuries were not evaluated separately. If the cases were analyzed separately using the proposed method, the discriminative power obtained when using penetrating injuries would be higher than that when using blunt injuries (AUC 0.982 vs 0.965). For different injury mechanisms, the results can be calculated using the same formula without the need to design another method. In this study, the data available in the trauma registry can be used to calculate the probability of death or survival from trauma for each patient by using a computer applet.

Among the methods that apply regression to evaluate the severity of trauma, AIS predot code-based assessment methods, such as TMPM and IMP, yield more accurate prediction results than those of ICD code-based methods, such as TMPM-ICD9, TMPM-ICD10, and IMP-ICDX.^[8,9,19–21]^ However, they are all based on the assessment of pure anatomical injuries and do not reflect the contribution of other available clinical information in the results. We speculate that the results of TRIMP-ICDX are similar to the comprehensive evaluation results based on IMP because adding other available information can correct the defects of IMP-ICDX. Therefore, the ICD-10-CM injury diagnostic code should be able to replace the AIS predot code. This approach requires considerable human and material resources as trauma surgery experts need to specifically set AIS predot codes when collecting data, which is difficult even for developed countries and more so for developing ones.

## 5. Limitations

In this study, NTDB data were used in the evaluation. The data were sourced from different hospitals and regions in the United States, and the setting of the ICD-10-CM code might have led to artificial differences. The results of this study may only be applicable to the data of different hospitals in the United States rather than all over the world because we apply full code of ICD-10-CM as the calculation unit (World Health Organization (WHO) standard code plus supplementary code), not the WHO certified standard ICD-10 code (i.e., the first 4 digit codes). What is more, the NTDB data are not population-based, thus, differences in data inevitably affected the results. The TRIMP-ICDX is based on the severity of anatomical injury as well as reference to more than 10 available variables, and finally evaluated by a separate logistic regression. Although there are many variables, these information is easily available in the clinic, and furtheermore, the evaluation process is simple, and the results are convincing. Moreover, interested parties will be allowed to further verify the results of this study.

## 6. Conclusions

In summary, as a comprehensive evaluation method based on the ICD-10-CM lexicon, TRIMP-ICDX is significantly better than IMP-ICDX and TRISS with respect to both discriminative power and calibration. The TRIMP-ICDX should become a research method for the comprehensive evaluation of trauma severity.

## Author contributions

MDW and GHZ contributed to the study concept and design. MDW, GHZ, and DGC contributed to the analysis and interpretation of data. MDW contributed to the acquisition of data. All authors contributed to the critical revision of the manuscript for important intellectual content. MDW and GHZ contributed to the drafting of the manuscript. YJZ and WHF contributed to the literature search. All authors read and approved the final manuscript.

## Acknowledgments

We gratefully acknowledge the assistance from American College of Surgeons for providing data set of the National Trauma Data Bank.

## Supplementary Material



## Footnotes

* Certification status based on American College of Surgeons and state designation.

† Results are presented as mean (SD).

* ln indicates natural logarithm.
