# Supplementary material for: Traumatic injury mortality prediction (TRIMP-ICDX): A new comprehensive evaluation model according to the ICD-10-CM codes
Source: Medicine (Baltimore). 2022 Aug 5;101(31):e29714. doi: 10.1097/MD.0000000000029714 (PMC9351923; doi:10.1097/MD.0000000000029714)
Supplement: Supplementary file 1 [file medi-101-e29714-s001.pdf]

Supplemental Digital Content（Appendix1）

Setting the coded value of each variable

In this paper, for multi-category variables, the corresponding code values are set according to different mortality rates, as shown in Table A. For each binary classification variable (such as ICU admission, mechanical ventilation, and surgical operation), the corresponding coded values are set as 1 for presence and 0 for absence.

Table A. The coded value of each variable

Coded value	SBP	RR	HR	Injury mechanism
1	100-174	15-24	60-100	Stabbing
2	>174	25-29	101-129	Violence *
3	75-99	10-14	40-59	Blunt injury
4	50-74	>29	>129	MVC
5	0-49	0-9	0-39	Fall
6				Gunshot

* Violence means to strike or against.

MVC Motor vehicle crash, *HR* Heart rate, *RR* Respiratory rate, *SBP* Systolic blood pressure.

Supplemental Digital Content（Appendix2）

Assessment of TRIMP-ICDX and *P*s

In order to evaluate TRIMP-ICDX, it is necessary to replace the ICD-10-CM codes with their respective WMDP values (see Additional file 4.xls of reference 9). The TRIMP_ICDX comprehensively integrated the severity of anatomical injury (such as WMDP and NBR based on the ICD-10-CM code), physiological reserve indicators (such as age, gender, and SCWI), and physiological response indicators to trauma (Whether to stay in ICU, mechanical ventilation, surgical operation, GCS, and vital signs) as basic predictors. And establish an independent logistic regression model. In order to reduce the difference in the model: continuous variables (such as NBR and age) were suggested by fractional polynomial analysis [13], binary indicator variables (such as ICU admission, mechanical ventilation, and surgical operation), and multi-category variables (including SBP, RR, HR, and injury mechanism) (see Appendix 1). The mathematical expressions were as follows:

$$
\begin{aligned}
TRIMP\_ICDX = \Lambda\Bigg(& C_0 + \sum_{r=1}^{5} C_r \times I_r + \sum_{s=6}^{8} C_s \times I_{s-5}{}^3 + C_9 I_1 \times I_2 + C_{10} S + C_{11} \sum_{i=1}^{50} NBR_i \\
& + C_{12} \sum_{i=1}^{50} \ln(NBR_i) + C_{13} age^3 + C_{14} age^3 \ln(age) + C_{15} gender + C_{16} SCWI \\
& + C_{17} ICU + C_{18} ventilator + C_{19} operation + C_{20} \sum_{j=1}^{6} mechanism_j \\
& + C_{21} \sum_{k=3}^{15} GCS_k + C_{22} \sum_{l=1}^{5} SBP_l + C_{23} \sum_{m=1}^{5} HR_m + C_{24} \sum_{n=1}^{5} RR_n \Bigg)
\end{aligned}
$$

where C_0 is constant and C_n are the corresponding regression coefficients for each variable, respectively, as shown in Table 3. Each variable in the above formula is a

binary indicator variable, no matter how many values it may contain. Ln is a function of the natural logarithm base *e*. Λ is the cumulative distribution function.

To reflect the actual impact of the survival probability (*P*s) of each patient. Finally TRIMP-ICDX is similar to TRISS [14-16] and converted to *P*s. The calculation formula is as follows:

$$Ps = \frac{1}{1 + Exp(TRIMP\_ICDX)}$$

Exp() is an exponential function of base *e* (*e* = 2.71828…).

With the popularity of computers, the above calculation method is easy for clinicians.

For example: Inc_key = 160909929, representing a 57-year-old male patient admitted to a Level-I trauma center in 2016 due to motor vehicle accident trauma (the coded value is set to 4, the same below), with a total of 9 injured body regions, ISS = 29. Vital signs upon arrival at hospital: SBP = 162 mmHg (coded value is 1), HR = 101 /min (coded value is 2), and RR = 18 /min (1), was admitted to ICU (1), mechanical ventilation (1) and no surgery on admission (0). The GCS score was 15, SCWI was 2 (Bleeding disorder and drug use disorder). Eventually the patient was hospitalized for 31 days and survived. The survival probability of each score is as follows: TRIMP-ICDX = -3.17319, *P*s = 0.9598 (that is 95.98%); IMP-ICDX = -3.42966 (*P*s = 0.9686); TRISS = 1.72489 (*P*s = 0.8488).
